# Validation of the T86I mutation in the *gyrA* gene as a highly reliable real time PCR target to detect Fluoroquinolone-resistant *Campylobacter jejuni*

**DOI:** 10.1186/s12879-020-05202-4

**Published:** 2020-07-16

**Authors:** Nereyda Espinoza, Jesús Rojas, Simon Pollett, Rina Meza, Lilian Patiño, Manuel Leiva, Máximo Camiña, Manuela Bernal, Nathanael D. Reynolds, Ryan Maves, Drake H. Tilley, Matthew Kasper, Mark P. Simons

**Affiliations:** 1 Bacteriology Department, U.S Naval Medical Research Unit-6 (NAMRU-6), Avenida Venezuela, Cuadra 36, Callao, Peru; 2 Instituto Nacional de Salud del Niño, Lima, Peru; 3 Hospital Nacional Docente Madre-Niño San Bartolomé, Lima, Peru; 4 Hospital de Emergencias Pediátricas, Lima, Peru; 5 Naval Medical Research Center, Silver Springs, MD USA; 6 Naval Medical Center San Diego, San Diego, California USA

**Keywords:** *Campylobacter jejuni*, T86I, *gyrA*, Ciprofloxacin, Antimicrobial resistance

## Abstract

**Background:**

*Campylobacter jejuni* is a leading cause of bacterial diarrhea worldwide, and increasing rates of fluoroquinolone (FQ) resistance in *C. jejuni* are a major public health concern. The rapid detection and tracking of FQ resistance are critical needs in developing countries, as these antimicrobials are widely used against *C*. *jejuni* infections. Detection of point mutations at T86I in the *gyrA* gene by real-time polymerase chain reaction (RT-PCR) is a rapid detection tool that may improve FQ resistance tracking.

**Methods:**

*C. jejuni* isolates obtained from children with diarrhea in Peru were tested by RT-PCR to detect point mutations at T86I in *gyrA*. Further confirmation was performed by sequencing of the *gyrA* gene.

**Results:**

We detected point mutations at T86I in the *gyrA* gene in 100% (141/141) of *C. jejuni* clinical isolates that were previously confirmed as ciprofloxacin-resistant by E-test. No mutations were detected at T86I in *gyrA* in any ciprofloxacin-sensitive isolates.

**Conclusions:**

Detection of T86I mutations in *C*. *jejuni* is a rapid, sensitive, and specific method to identify fluoroquinolone resistance in Peru. This detection approach could be broadly employed in epidemiologic surveillance, therefore reducing time and cost in regions with limited resources.

## Background

*Campylobacter* infection is the leading zoonotic cause of foodborne illness in the world, with 400 million acute cases of diarrheal disease reported worldwide annually [1]. *C. jejuni* is a leading bacterial cause of human diarrheal infections [2, 3] in high income countries, but the burden of campylobacteriosis is markedly elevated in low-to-middle income countries (LMIC) and contributes significantly to childhood mortality and poor linear growth in these regions [1, 4–6]. In addition to potentially severe dysentery, campylobacteriosis may also result in longer-term secondary complications including Guillain-Barre syndrome and reactive arthritis [1]. Antibiotics usually considered for treatment of campylobacteriosis include macrolides and fluoroquinolones (FQs) due to their relatively low cost, limited short-term side effects and ease of administration [7].

Globally, the emergence of FQ resistance in *C. jejuni* has become a major problem in treating undifferentiated dysentery and campylobacteriosis [7–10]. FQs, including ciprofloxacin, are among the most widely used antibiotics for the treatment of travelers’ diarrhea. However, increasing rates of resistance to these drugs have been reported worldwide [11], with significant treatment implications for the management of *Campylobacter* infections in developing settings with limited access to resources and alternative therapeutic choices [12–14].

The antibacterial activity of ciprofloxacin and other FQs is due to their ability to bind and inhibit the DNA gyrase necessary for genomic DNA replication. Resistance to ciprofloxacin is mediated by mutations in the *gyrA* gene, with the single-step T86I amino acid change as one of the most common mutations associated with decreased susceptibility in *Campylobacter* [1, 7–10]. Alterations of nucleotide 257 (codon 86) from ACA to ATA of the *gyrA* gene result in a threonine to isoleucine substitution in the GyrA protein and confer high- level resistance to ciprofloxacin [15–20].

Numerous molecular techniques based on polymerase chain reaction (PCR) testing may detect mutations associated with resistance to FQ and include sequencing, detection of polymorphisms, and denaturing gradient gel electrophoresis [9, 16, 21, 22]. In recent years, several of these genotypic methods for the detection of FQ-resistant *Campylobacter* have been validated. To date, all have identified the T86I mutation through nucleic acid amplification [17, 23–25]. However, there are few validation studies among *Campylobacter* isolates from LMICs where rates of antimicrobial resistance (AMR) are particularly high [26, 27].

The primary goal of this study was to evaluate a RT-PCR assay for detection of the T86I *gyrA* gene mutation among *C. jejuni* isolates from Peru, a middle-income country with rapidly rising rates of FQ-resistant *Campylobacter* [27] and a substantial burden of campylobacteriosis [6, 26–28].

## Methods

### Study sites

A surveillance study for AMR in enteric pathogens in Peru occurred between January 2001 to December 2010 at nine hospital and community laboratories in the cities of Lima, Cusco, and Iquitos [27]. Stool isolates identified as *Campylobacter* spp. were placed in Cary-Blair transport media and stored at 4 °C at the study site [27]. Every two weeks, isolates from each study site were transported to the U.S. Naval Medical Research Unit No. 6 (NAMRU-6) laboratory at 4 °C for further testing. After microbiological confirmation using routine culture and biochemical methods, all isolates were stored at − 80 °C in Peptone Broth with 15% glycerol.

A random selection of 200 archived stool isolates from children between 1 month to 15 years old during period 2006–2010 were selected from three hospitals in Lima, Peru (Instituto Nacional de Salud del Niño, Hospital Nacional Docente Madre-Niño, and Emergencias Pediatricas) for antimicrobial susceptibility testing and detection of T86I *gyrA* mutation.

### Culture and isolation of C. jejuni

All archived isolates that were previously identified as hippurate hydrolysis-positive were re-cultured and re-isolated according to methods described elsewhere [27] for further testing.

### Extraction of C. jejuni DNA

Isolates were reactivated on Columbia agar (Becton, Dickinson and Company, Sparks, MD 21152 USA) that was supplemented with 5% sheep blood after incubation for 48 h at 42 °C under micro-aerobic conditions. One loop of colony growth was then used for DNA extraction which was performed by QIAamp DNA Mini Kit (Qiagen GmbH, D40724 Hilden, Germany), following the manufacturer’s guidelines. The final concentration of DNA was adjusted to 10 ng/μl.

### Molecular confirmation of C. jejuni species

*C. jejuni* isolates underwent further species confirmation by PCR. Primers designed by Vandamme et al. [29] were used to amplify internal *glyA* fragments and detect a 773-bp amplification product consistent with a *C. jejuni* species (Table 1). For PCR reaction, 11 μl of AmpliTaq Gold PCR Master Mix 2X, (AB Applied Biosystems, Foster City, CA 94404, USA), 2 μl of DNA [10 ng/ μl] and primers at final concentration of 0.4 μM.DNA were mixed for a final volume of 25 μl. Amplification was performed by Veriti Thermal Cycler (AB Applied Biosystems, Singapore) using the parameters: initial denaturation (5 min 95 °C), twice (1 min 94 °C, 1 min 64 °C, 1 min 72 °C), twice (1 min 94 °C, 1 min 62 °C, 1 min 72 °C), twice (1 min 94 °C, 1 min 60 °C, 1 min 72 °C), twice (1 min 94 °C, 1 min 58 °C, 1 min 72 °C)), twice (1 min 94 °C, 1 min 56 °C, 1 min 72 °C) 30 cycles (1 min 94 °C, 1 min 54 °C, 1 min 72 °C) and final extension step 10 min at 72 °C as recommended by Vandamme et al. [29]. Post-amplification, PCR products were visualised by 2% UltraPure agarose gel (Invitrogen, Carlsbad, CA 92008, USA) with a SYBR Safe stain (1X) (Invitrogen, Eugene, Oregon, 541.4658300, USA).

**Table 1:** Primers and probes used

Target gene	Oligonucleotide sequence (5′-3′)	Reference
*glyA*	CATCTTCCCTAGTCAAGCCT	Vandamme, et al. [29]
	AAGATATGGCTCTAGCAAGAC	
*gyrA*	TGCTGTTATAGGTCGTTA	This study
	CCTTGTCCTGTAATACTTG	
*gyrA* Wild type	HEX 5′-CCCACATGGAGATACAGCAGTTTATG-3′-BHQ2	This study
*gyrA* point mutation T86I	6-FAM 5’CCCACATGGAGATATAGCAGTTTATG-3’BHQ1	This study
*gyrA*	GCCTGACGCAAGAGATGGTT	Bakeli, et al. [17]
	TTTGTCGCCATACCTACAGC	

### Determination of phenotypic resistance

Positively-identified *C. jejuni* isolates were tested for antibiotic susceptibility using the E-test to determine the minimum inhibitory concentration (MIC) for ciprofloxacin at a range of 0.002-32 μg/mL (AB BioMérieux, bioMérieux SA RCS LYON 69280 Marcy-l’Etoile, France) [30]. Isolates also underwent disk-diffusion testing [27] using Clinical and Laboratory Standards Institute (CLSI) guidelines to determine susceptibility [31]. *C. jejuni* ATCC 33560 was used as the quality control organism (American Type Culture Collection, Manassas, Virginia, USA).

### Determination of genotypic resistance

A blinded molecular detection of antimicrobial susceptibility was performed on all isolates to generate unbiased results. Isolates were tested for the presence of the T86I *gyrA* resistance mutation using real-time PCR. Specific TaqMan probes and primers (Table 1) were designed by AlleleID 7 software (PREMIER Biosoft International, Palo Alto, California, USA). For a final volume of 20 μl for the PCR reaction, the following were used: 10 μl of Rotor Gene Multiplex PCR (QIAGEN GmbH, Hilden, Germany), 1 μl of DNA [10 ng/ μl,] primers, and probes at final concentration of 0.5 μM and 0.25 μM respectively.

DNA amplification and the resultant PCR product was analysed using the Rotor Gene Q version 1.7.94 (QIAGEN GmbH, Hilden, Germany), using the following parameters: pre-denaturation at 95 °C for 5 min, 40 cycles of denaturation at 95 °C for 10 s, annealing at 55 °C for 30 s, and extension at 72 °C for 20 s with acquisition in green and yellow channels.

### Detection of ciprofloxacin resistance for allelic discrimination

Detection of T86I point mutation in the *gyrA* gene of *C. jejuni* was carried out by allelic discrimination. A fluorescent signal from only the HEX dye with acquisition in the yellow channel indicated detection of the wild type (mutation-negative) gene, while the presence of 6-FAM dye fluorescence with acquisition in green channel indicated detection of the T86I mutation (mutation-positive). Absence of both fluorescent signals indicated the gyrA gene was not amplified as observed with *C. jejuni* in no-template controls (NTCs) and *C. coli* isolates. For discrimination of positive and negative mutants, we used a wild-type control (ATCC 33560), a T86I mutant that was identified by sequencing the *gyrA* gene, and a non-template controls for each run, with a threshold of 0.1 for genotypic discrimination. Fluorescent signals were interpreted automatically using sequence detection software using the Rotor Gene Q version 1.7.94 (QIAGEN GmbH, Hilden, Germany).

### Validation of T86I gyrA real-time PCR assay diagnostic performance

Sensitivity and specificity were determined for the T86I *gyrA* resistance mutation real-time PCR assay using disk-diffusion and E-test as gold standards. Confidence intervals (95%) for sensitivity and specificity were determined without continuity correction [32, 33].

### Detection of amino acid mutations in gyrA to confirm PCR results in a subset of isolates

Sequencing of the amplified fragment of *gyrA* gene from a random subset of 100 of the 189 *C. jejuni* study isolates were performed to confirm the specific point amino acid mutation T86I in the sequence by, using primers (Table 1) and PCR conditions already described [17], except that the pre-denaturation time was 1 min and the annealing time was 5 s.

### Ethical considerations

The Institutional Review Board of the U.S. Naval Medical Research Unit 6 in Peru (FWA 00010031) determined that the testing of these bacterial isolates (Protocol No PJT.NMRCD.HURS03) did not constitute human subjects research.

## Results

Of the 200 *Campylobacter* isolates, 189 were confirmed as *C. jejuni* and 11 as *C. coli* using biochemical and molecular techniques [29]. Only the 189 *C. jejuni* isolates were used for the validation study and underwent antimicrobial susceptibility testing.

Phenotypic detection of AMR identified 74.6% (141/189) of the *C. jejuni* isolates as resistant to ciprofloxacin and naldixic acid by disk-diffusion and E-test (Table 2). Further, the molecular detection of the T86I *gyrA* mutation was observed in 100% (141/141) of ciprofloxacin-resistant phenotypes and none of the ciprofloxacin-sensitive phenotypes (Table 3). The T86I *gyrA* mutation detection assay thus demonstrated a sensitivity of 100% (95% CI 97.35–100%) and a specificity of 100% (95% CI 92.6–100%) in comparison to conventional antimicrobial susceptibility testing techniques. Moreover, this assay correctly identified all *C. jejuni* isolates in addition to the ciprofloxacin resistant/sensitive phenotypes, while no *C*. *coli* isolates were detected.

**Table 2:** Susceptibility of 189 *C. jejuni* isolates, and point mutation T86I of the *gyrA* gene

Phenotype	No. of Isolates	CIP_Disk diffusion	NA_Disk diffusion	CIP_MIC	Real-Time PCR
		Range (mm)	Range (mm)	Range (μg/mL)	***gyrA*** gene (n)
CIP-S and NA-S	44	29–50	19–37	0.008–1.0	Wild type 44
CIP-R and NA-R	141	6–11	6–9	4–32	T86I mutation 141
CIP-S and NA-R	4	24–38	6	0.047–0.19	Wild type 4

*CIP-S* ciprofloxacin sensitive, *CIP-R* ciprofloxacin resistant, *NA-S* nalidixic acid sensitive, *NA-R* nalidixic acid resistant, *MIC* minimal inhibitory concentration

**Table 3:** Comparison of Real Time PCR results with ciprofloxacin antimicrobial susceptibility by E-Test

Real Time PCR T86I	Total no. of isolates CIP_MIC_Sensitive	Total no. of isolates CIP_MIC_Resistant
Wild type (48)	48	0
Mutation (141)	0	141

Seventy-two of the *C. jejuni* isolates that were positive with the T86I PCR assay and 28 of negative wild-type strains were further selected for DNA sequencing of *gyrA* as previously described [17]. The sequencing results of *gyrA* mutant and wild-type isolates are presented in Table 4. All 72 *C. jejuni* isolates demonstrated amino-acid substitutions at codon 86 (T → I). None of the 28 wild-type isolates demonstrated a point mutation at *gyrA* nucleotide position 257. The amino-acid substitution V149I was also observed in 28 ciprofloxacin resistant isolates as previously reported, with none of the ciprofloxacin-susceptible isolates showing this change. Table 5 shows the distribution of *C. jejuni* isolate by hospital and by year, as well as the percentage of those that carried the T86I *gyrA* mutation from 2006 to 2010. Overall, the prevalence of the gene mutation appeared in 65–75% of all isolates for each year until 2010, when it was identified in 100% of all isolates tested.

**Table 4:** Correlation between Real-Time PCR T86I mutation MICs values of ciprofloxacin and mutations of the *gyrA* gene

Real Time PCR Mutation T86I	Strains (***n*** = 100)	CIP_MIC (μg/mL)	T86I	A120V	D144N	V149I	H81	G110	S119	Y152	S157	V161
			ACA	GCC	GAT	GTT	CAC	GGC	AGT	TAT	AGC	GTT
			ATA	GTC	AAT	ATT	CAT	GGT	AGC	TAC	AGT	GTC
Positive	28	12–32	T	T	–	–	T	–	C	–	T	C
Positive	28	12–32	T	T	–	A	T	–	C	–	T	C
Positive	7	32	T	T	–	–	T	T	C	–	T	C
Positive	3	12–32	T	T	–	–	T	–	C	C	T	C
Positive	6	6–32	T	–	–	–	–	–	–	–	–	–
Negative	21	0.016–0.190	–	T	–	–	T	–	C	–	T	C
Negative	1	0.064	–	T	–	–	T	T	C	–	T	C
Negative	1	0.032	–	T	A	–	T	–	C	–	T	C
Negative	5	0.023–0.047	–	–	–	–	–	–	–	–	–	–

**Table 5:** Real time PCR detection of ciprofloxacin sensitive or wild type (WT) *C. jejuni* isolates compared to resistant or T86I positive *C. jejuni* isolates per year by hospital

Hospital	2006		2007		2008		2009		2010	
	T86I (%)	WT	T86I (%)	WT	T86I (%)	WT	T86I (%)	WT	T86I (%)	WT
Hospital Materno Infantil	11 (92)	1	9 (75)	3	1 (25)	3	4 (100)	0	9 (100)	0
Emergencias Pediátricas	12 (63)	7	9 (53)	8	13 (68)	6	13 (65)	7	12 (100)	0
Hospital del Niño	9 (75)	3	16 (76)	5	11 (92)	1	3 (43)	4	9 (100)	0
**Total**	**32(74)**	**11**	**34(68)**	**16**	**25(72)**	**10**	**20(65)**	**11**	**30(100)**	**0**

## Discussion

Increasing AMR in *C. jejuni* is a major global public health threat. It is important to seek alternatives to conventional phenotypic testing of AMR in *C. jejuni* due to the limited availability of resources to isolate and characterize enteric pathogens in developing countries. This study is one of only a few studies that have validated this method in LMICs, including Latin America.

We have previously described increasing rates of AMR in *Campylobacter* isolates in Peru [27]. Our data in this study suggest that an increased prevalence of the T86I *gyrA* mutation in *C. jejuni* may be the cause of this rise in FQ resistance (Table 5). The very high sensitivity (100%) and specificity (100%) demonstrated by T86I *gyrA* mutation real-time PCR assay confirms it as a valid alternative resistance assay to conventional fluoroquinolone susceptibility testing in *C. jejuni* isolates, consistent with the high sensitivity and specificity described elsewhere [15, 16, 18]. This data set may shed light upon the feasibility of this technique to be broadly employed in other regions for detecting changes in ciprofloxacin resistance among *C. jejuni*. Although there are other mutations associated with ciprofloxacin resistance in *gyrA* gene of *C*. *jejuni*, the T86I mutation is the most frequent mutation encountered worldwide [17]. A different mutation at the same codon, T86A, has also been described in *C. jejuni* isolates with resistance to nalidixic acid [16–18], but its association with resistance to later-generation FQs such as ciprofloxacin is less clear. The present study found no T86A mutations in *C*. *jejuni* isolates through *gyrA* sequencing.

In these Peruvian isolates, the T86I substitution was the main amino-acid change associated with high-level resistance to ciprofloxacin, as described in other settings [9, 16, 17, 22, 28]. Nonetheless, these findings may be specific to given geographical regions with other common amino-acid substitutions (e.g., V149I) being associated with FQ resistance elsewhere [34]. However for some of these amino-acid substitutions their relevance as a mediator of AMR have been placed into question with their presence being found in sensitive *C. jejuni* isolates as well as being absent in a large proportion of FQ-resistant isolates [17, 28]. Until additional characterization studies are performed, questions will remain about the significance of these other mutations in the epidemiology of AMR in *Campylobacter*.

Overall, this technique offers an alternative, reliable, and rapid means for detecting FQ resistance in *C. jejuni* isolates, thus avoiding more complex susceptibility testing for this fastidious microorganism*.* With the recent alarming rise in FQ-resistant *Campylobacter* in Peru [6, 27, 35], this assay may serve as a useful tool for quality control of conventional *Campylobacter* antibiotic resistance testing, such as disk-diffusion or E-test, in both clinical care and in AMR surveillance.

We utilized E-test strips instead of broth microdilution for confirmatory susceptibility testing, as previously validated [36]. A limitation of our study was the lack of isolates with intermediate susceptibility to FQs, for which the detection of a molecular determinant of resistance may be useful for antibiogram interpretation, particularly in instances where isolates are non-susceptible to alternative antimicrobials such as macrolides, which has been noted in Peru [27]. Nonetheless, given the high sensitivity and specificity of the results herein presented, this assay could be a useful tool for the correct identification of *C. jejuni* as well as detection of FQ-resistant isolates from stool samples in developing countries, potentially saving time and money. Additionally, this assay may be applied as a reference control for traditional AMR methods in LMIC where capacities are limited. Given the clinical consequences of this emerging multi-drug resistant phenotype in low-middle income countries, further studies could address the validity and feasibility of molecular methods that simultaneously detect the mutations associated with resistance to both quinolones and macrolides. Effective molecular diagnostics may be a feasible approach to guide the antimicrobial treatment of *C. jejuni* infections.

## Conclusions

The present study developed a rapid, sensitive, and specific molecular technique for the detection of T86I mutations in the *gyrA* gene of *C*. *jejuni* that appears strongly associated with FQ resistance. Because of the high prevalence of *C*. *jejuni* in LMIC and the concern for increasing FQ resistance, this detection approach could be broadly employed in epidemiologic surveillance to reduce time and cost in regions with limited resources.

## Data Availability

The datasets used and/or analyzed during the current study are available from the corresponding author on reasonable request.

## Abbreviations

FQ: Fluoroquinolone
RT-PCR: Real time polymerase chain reaction
LMIC: Low-to-middle income countries
PCR: Polymerase chain reaction
NAMRU-6: Naval Medical Research Unit No. 6
MIC: Minimum inhibitory concentration
CLSI: Clinical and Laboratory Standards Institute
CIP-R: Ciprofloxacin resistant
NA-R: Nalidixic acid resistant
AMR: Antimicrobial resistance

## References

[1] Nachamkin I, Szymanski CM, Blaser MJ (2008). Campylobacter.

[2] Allos BM (2001). Campylobacter jejuni infections: update on emerging issues and trends. Clin Infect Dis.

[3] Kirkpatrick BD, Tribble DR (2011). Update on human campylobacter jejuni infections. Curr Opin Gastroenterol.

[4] Lee G, Paredes Olortegui M, Peñataro Yori P, Black RE, Caulfield L, Banda Chavez C (2014). Effects of Shigella-, campylobacter- and ETEC-associated diarrhea on childhood growth. Pediatr Infect Dis J.

[5] Francois R, Yori PP, Rouhani S, Salas MS, Olortegui MP, Trigoso DR, Pisanic N, Burga R, Meza R, et al. The other Campylobacter: Not innocent bystanders in endemic diarrhea and dysentery in children in low-income settings. PLOS Neglected Tropical Dis. 2018;12(2):e0006200. 10.1371/journal.pntd.0006200 PMC5819825 29415075

[6] Lee G, Pan PW, Pablo P, Maribel P, Drake T, Michael G, Richard O, Rosa B, Cesar B, Margaret K. Symptomatic and Asymptomatic Campylobacter Infections Associated with Reduced Growth in Peruvian Children. PLOS Neglected Tropical Dis. 2013;7(1):e2036. 10.1371/journal.pntd.0002036 PMC3561130 23383356

[7] Alfredson DA, Korolik V (2007). Antibiotic resistance and resistance mechanisms in campylobacter jejuni and campylobacter coli. FEMS Microbiol Lett.

[8] Engberg J, Aarestrup FM, Taylor DE, Gerner-Smidt P, Nachamkin I (2001). Quinolone and macrolide resistance in campylobacter jejuni and C. coli: resistance mechanisms and trends in human isolates. Emerg Infect Dis.

[9] Niwa H, Chuma T, Okamoto K, Itoh K (2003). Simultaneous detection of mutations associated with resistance to macrolides and quinolones in campylobacter jejuni and C. coli using a PCR-line probe assay. Int J Antimicrob Agents.

[10] Fabrega A, Sanchez-Cespedes J, Soto S, Vila J (2008). Quinolone resistance in the food chain. Int J Antimicrob Agents.

[11] Taylor DN, Sanchez JL, Candler W, Thornton S, McQueen C, Echeverria P (1991). Treatment of travelers' diarrhea: ciprofloxacin plus loperamide compared with ciprofloxacin alone. A placebo-controlled, randomized trial. Ann Intern Med.

[12] Holloway K, Mathai E, Gray A (2011). Surveillance of antimicrobial resistance in resource-constrained settings - experience from five pilot projects. Tropical Med Int Health.

[13] Holloway K, Mathai E, Gray A (2011). Surveillance of community antimicrobial use in resource-constrained settings--experience from five pilot projects. Tropical Med Int Health.

[14] Kotwani A, Holloway K (2011). Trends in antibiotic use among outpatients in New Delhi, India. BMC Infect Dis.

[15] Said MM, El-Mohamady H, El-Beih FM, Rockabrand DM, Ismail TF, Monteville MR (2010). Detection of gyrA mutation among clinical isolates of campylobacter jejuni isolated in Egypt by MAMA-PCR. J Infect Dev Ctries.

[16] Dionisi AM, Luzzi I, Carattoli A (2004). Identification of ciprofloxacin-resistant campylobacter jejuni and analysis of the gyrA gene by the LightCycler mutation assay. Mol Cell Probes.

[17] Bakeli G, Sato K, Kumita W, Saito R, Ono E, Chida T (2008). Antimicrobial susceptibility and mechanism of quinolone resistance in campylobacter jejuni strains isolated from diarrheal patients in a hospital in Tokyo. J Infect Chemother.

[18] Kinana AD, Cardinale E, Bahsoun I, Tall F, Sire JM, Garin B (2007). Analysis of topoisomerase mutations in fluoroquinolone-resistant and -susceptible campylobacter jejuni strains isolated in Senegal. Int J Antimicrob Agents.

[19] Oporto B, Juste RA, Hurtado A (2009). Phenotypic and genotypic antimicrobial resistance profiles of campylobacter jejuni isolated from cattle, sheep, and free-range poultry Faeces. Int J Microbiol.

[20] Han J, Wang Y, Sahin O, Shen Z, Guo B, Shen J (2012). A fluoroquinolone resistance associated mutation in gyrA affects DNA supercoiling in campylobacter jejuni. Front Cell Infect Microbiol.

[21] Zirnstein G, Li Y, Swaminathan B, Angulo F (1999). Ciprofloxacin resistance in campylobacter jejuni isolates: detection of gyrA resistance mutations by mismatch amplification mutation assay PCR and DNA sequence analysis. J Clin Microbiol.

[22] Wang Y, Huang WM, Taylor DE (1993). Cloning and nucleotide sequence of the campylobacter jejuni gyrA gene and characterization of quinolone resistance mutations. Antimicrob Agents Chemother.

[23] Beckmann L, Muller M, Luber P, Schrader C, Bartelt E, Klein G (2004). Analysis of gyrA mutations in quinolone-resistant and -susceptible campylobacter jejuni isolates from retail poultry and human clinical isolates by non-radioactive single-strand conformation polymorphism analysis and DNA sequencing. J Appl Microbiol.

[24] Chatzipanagiotou S, Papavasileiou E, Lakumenta A, Makri A, Nicolaou C, Chantzis K (2002). Antimicrobial susceptibility patterns of campylobacter jejuni strains isolated from hospitalized children in Athens, Greece. J Antimicrob Chemother.

[25] Sonnevend A, Rotimi VO, Kolodziejek J, Usmani A, Nowotny N, Pal T (2006). High level of ciprofloxacin resistance and its molecular background among campylobacter jejuni strains isolated in the United Arab Emirates. J Med Microbiol.

[26] Oberhelman RA, Gilman RH, Sheen P, Cordova J, Zimic M, Cabrera L (2006). An intervention-control study of corralling of free-ranging chickens to control campylobacter infections among children in a Peruvian periurban shantytown. Am J Trop Med Hyg.

[27] Pollett S, Rocha C, Zerpa R, Patino L, Valencia A, Camina M (2012). Campylobacter antimicrobial resistance in Peru: a ten-year observational study. BMC Infect Dis.

[28] Luque A, Riveros M, Prada A, Ochoa T, Ruiz J. Virulence and Antimicrobial Reistance in Campylobacter spp. from a Peruvian Pediatric Cohort. Hindawi Scientifica. 2017;2017:7848926. 10.1155/2017/7848926 PMC5654289 29130018

[29] Vandamme P, Van Doorn LJ, al Rashid ST, Quint WG, van der Plas J, Chan VL (1997). Campylobacter hyoilei Alderton et al. 1995 and campylobacter coli Veron and Chatelain 1973 are subjective synonyms. Int J Syst Bacteriol.

[30] Baker CN (1992). The E-test and campylobacter jejuni. Diagn Microbiol Infect Dis.

[31] CLSI Methods for Antimicrobial Dilution and Disk Susceptibility Testing of Infrequently Isolated or Fastidious Bacteria`; Approved Guidelines - 2nd Edition. CLSI document M45-A2. Wayne, PA. 2016.

[32] VassarStats: Website for Statistical Computation. Statisticalhttp://vassarstats.net/prop1.html. Accessed 21 Oct 2014.

[33] Newcombe RG (1998). Two-Sided Confidence Intervals for the Single Proportion: Comparison of Seven Methods. Statistics Med.

[34] Ge B, White DG, McDermott PF, Girard W, Zhao S, Hubert S (2003). Antimicrobial-resistant campylobacter species from retail raw meats. Appl Environ Microbiol.

[35] Fernandez H (2011). Campylobacter and campylobacteriosis: a view from South America. Rev Peru Med Exp Salud Publica.

[36] Luber P, Bartelt E, Genschow E, Wagner J, Hahn H. Comparison of Broth microdilution, E Test, and agar dilution methods for antibiotic susceptibility testing of Campylobacter jejuni and Campylobacter coli. J Clin. Microbiol. 2003;41(3):1062–8. 10.1128/JCM.41.3.1062-1068.2003 PMC150256 12624030

